# Variations in rumen microbiota and host genome impacted feed efficiency in goat breeds

**DOI:** 10.3389/fmicb.2025.1492742

**Published:** 2025-01-29

**Authors:** Alaa Emara Rabee, Ibrahim Abou-souliman, Ahmed I. Yousif, Mebarek Lamara, Mohamed A. El-Sherbieny, Eman Ali Elwakeel, Ahmed M. Sallam

**Affiliations:** ^1^Animal and Poultry Nutrition Department, Desert Research Center, Cairo, Egypt; ^2^Animal and Poultry Breeding Department, Desert Research Center, Cairo, Egypt; ^3^Animal Production Research Institute, Agricultural Research Center, Cairo, Egypt; ^4^Forest Research Institute, University of Quebec in Abitibi-Temiscamingue, Rouyn-Noranda, QC, Canada; ^5^Department of Animal and Fish Production, Faculty of Agriculture, University of Alexandria, Alexandria, Egypt

**Keywords:** goat breeds, feed efficiency, caprine genome, rumen fermentation, bacteria and archaea

## Abstract

**Introduction:**

Improving feed efficiency (FE) is a significant goal in animal breeding programs. Variations in FE and its relationship with rumen microbiota remain poorly understood across different goat breeds.

**Methods:**

This study assessed the influence of the rumen microbiome and host genome on FE in 10 Shami (SH) goats and 10 Zaraibi (ZA) goats, all of which were fed the same diet. The animals were genotyped using the Illumina 65KSNP chip arrays *v2*, and their rumen bacteria and archaea were investigated using PCR-amplicon sequencing of the 16S rRNA gene.

**Results:**

The results showed that the ZA goats exhibited higher FE than the SH goats (*p* < 0.05) and a greater relative abundance (*p* < 0.05) of rumen bacterial groups that are involved in the degradation of cellulose and hemicelluloses, such as Bacteroidota and Fibrobacterota, along with genera such as *Prevotella*, Lachnospiraceae AC2044 group, Lachnospiraceae NK3A20 group, and *Succiniclasticum*, which are linked to carbohydrate and nitrogen metabolism pathways. In addition, breed differences were found to affect the proportions of milk fatty acids. The association analysis identified 26 genome windows containing several putative candidate genes, such as *TMEM241*, *AP4S1*, *FTO*, *HYAL2*, *BBS2*, *CD52*, *CRYBG2*, *PIGV*, *WDTC1*, *EEF1A2*, *GBA2*, *FNIP1*, *ACSL6*, *STARD10*, *VPS26B*, *ACAD8*, *GLB1L3*, *NRN1L*, *LCAT*, and *SLC7A6*. These genes contributed to FE traits in Egyptian goats, as they are involved in obesity, metabolism, and the transport of energy, vitamins, fatty acids, proteins, and lipids through diverse biological pathways.

**Discussion:**

This study suggests that specific genetic markers and rumen microbial traits could be used to identify high-efficient individuals in Egyptian goat breeds, and improving breeding strategies for FE.

## Introduction

Increased milk and meat production in ruminant animals, such as goats, is associated with the depletion of feed resources and methane (CH_4_) emissions, highlighting the importance of enhancing productivity while mitigating environmental impacts (Xue et al., 2022).

Feed efficiency (FE) is closely associated with microbial fermentation, which depends on high-efficient microbial groups, including bacteria, archaea, protozoa, and fungi (Li and Guan, 2017; Løvendahl et al., 2018).

These microbial groups work together to break down and transform the ingested animal diet into microbial protein (MP) and volatile fatty acids (VFAs), which fulfill the host animal’s energy and protein needs (Firkins and Yu, 2015). Also, microbial fermentation generates hydrogen (H_2_) and carbon dioxide (CO_2_) that are used, besides other substrates, such as acetic and formic acid and methanol, as substrates to produce methane by rumen archaea (Difford et al., 2018). Methane represents a loss in the animal’s gross energy intake and contribute to greenhouse gas emissions (Rabee et al., 2022).

Understanding the relationship between the rumen microbiome and FE can improve current breeding programs by facilitating the selection of highly efficient animals (Xue et al., 2022).

Additionally, recent studies demonstrated the association between the host genome and the rumen microbial community in cattle (Li et al., 2019). Therefore, effective strategies to improve FE and reduce methane emissions in livestock should focus on both animal genomes and rumen microbiomes (Difford et al., 2018).

Animals with higher FE exhibited lower methane emissions, a lower relative abundance of *Methanobrevibacter*, and higher levels of Candidatus Methanomethylophilus archeae and propionic acid-producing bacteria (Tapio et al., 2017; Bharanidharan et al., 2018; McLoughlin et al., 2020). Previous studies on rumen microbiota indicated that the bacterial community in goats was affiliated mainly with the phyla Bacteroidetes and Firmicutes, and the dominant genera were *Prevotella*, *Butyrivibrio*, and *Ruminococcus* (Giger-Reverdin et al., 2020; Luo et al., 2022), whereas the gut archaeal community was dominated by the genera *Methanobrevibacter* and *Methanosphaera* (Luo et al., 2022).

Feed constitutes the largest expense in livestock production, making selective breeding a key strategy to reduce costs (Singh et al., 2022; Khanal et al., 2023). Thus, identifying genetically superior animals with high FE has become integral to breeding programs. The recent advances in high-throughput sequencing technologies have helped breeders collect adjunct information about the genetic potentiality of their animals to be included in genomic selection (Weigel, 2017). The best animals (i.e., animals that eat less feed while maintaining similar production to their herd mates) are then selected for breeding to transmit their genes of high FE to the next generations (Khanal et al., 2023). Genome-wide association studies (GWAS) use hundreds of thousands of genetic markers, typically single-nucleotide polymorphisms (SNPs), that spread out across the entire genome to identify genetic markers for the trait of interest, which supports genomic selection in livestock (Desire et al., 2018; Moreira et al., 2019; Taussat et al., 2020; Barría et al., 2021; Uffelmann et al., 2021; Khanal et al., 2023).

Zaraibi (also known as Egyptian Nubian) and Shami (also known as Damascus) goats are the most important goat breeds in Egypt due to their high potential for fertility and milk production under harsh conditions (Eid et al., 2020; Almasri et al., 2023). Therefore, animals of the two breeds were involved in genetic improvement programs for exotic breeds in several countries (Güney et al., 2006; Tatar et al., 2021). The rumen microbiome and host genome of goat breeds in arid regions received less attention. Furthermore, few studies explored the association between the rumen microbiome, host genome, and FE in ruminant animals. Therefore, this study aimed to investigate the relationship between FE, rumen microbiota, and host genome in Shami and Zaraibi goat breeds.

## Materials and methods

### Ethics statement

All animal procedures included in the current study were approved by the Animal Breeding Ethics Committee at the Desert Research Center (DRC) in Egypt (reference number: AB/NO2022) and the Research Ethics Review Committee, Faculty of Agriculture, University of Alexandria, Egypt (Reference: Alex. Agri. 082305307). All methods and protocols in this study comply with the ARRIVE 2.00 guidelines. The experiment did not include animal euthanasia, and all animals were released to the goat herd after the end of the experiment.

### Animals and sampling

The experiment was conducted at the Agriculture Research Station, Animal Production Research Institute, Agricultural Research Center (ARC), Sakha, Kafr El Sheikh, Egypt. A total of 20 lactating goats were selected to represent both breeds, ensuring sufficient statistical power for comparative analysis. The animals comprised Shami goats (SH) with an average body weight of 40.6 ± 1.39 kg (*n* = 10) and Zaraibi goats (ZA) with an average body weight of 25.3 ± 1.57 kg (*n* = 10). The experiment lasted 45 days, with a 15-day adaptation period followed by 30 days of data collection. The adaptation period aimed to adapt the animals to individual housing conditions and feeding systems. All the animals used in this study were the offspring from the Sakha Agriculture Research Station goat herd in Kafr El Sheikh, Egypt. The body weight of the animals was recorded at the beginning of the experiment, and the animals were housed individually and had free access to drinking water. All animals in both groups received the same diet throughout the experiment: fresh Egyptian clover (*Trifolium alexandrinum*) *ad libitum* and commercial concentrates (2.5% of live weight per head/day). The quantities of offered and refused clover and concentrates feed mixture were estimated daily for each goat throughout the experiment. Subsequently, dry matter intake (DMI) was calculated as the difference between the offered and refused feed. After 20 days, approximately 200 mL of rumen fluid sample was collected from each doe via a stomach tube before morning feeding. The rumen fluid samples were filtered through a two-layer cheesecloth. The pH of rumen samples was measured using a digital pH meter (WPA CD70, ADWA, Szeged, Hungary). Then, the samples were separated into two portions, which were frozen at −20°C for DNA extraction and to analyze rumen ammonia (NH_3_-N) and volatile fatty acids (VFA). Milk yield was measured individually on days 20, 35, and 45 using twice-daily hand milking, morning and evening. Approximately 100 mL of representative milk samples were collected to conduct milk chemical composition. At the end of the experiment, all animals were released back to the goat herd without euthanasia.

### FE calculation

The gross FE was calculated using the equation FE = milk production (kg/d) divided by DMI (kg/d). Fat-corrected milk for 3.5% fat content was calculated using the formula: milk yield (MY) 3.5% = (0.432 + 0.1625 × % milk fat) × milk yield, kg/d (Sklan et al., 1992). Subsequently, adjusted FE was calculated using the formula: FE = 3.5% fat-corrected milk yield (kg)/dry matter intake (kg). Milk net energy (Milk NE) was calculated using the equation described in National Research Council (NRC) (2001): Milk NE (Mcal of NE_L/d) = Milk Production x (0.0929 * Fat % + 0.0563 * Protein +0.0395 * Lactose %). Finally, FE for lactation was calculated using the formula: Mcal/kg = Milk NE (Mcal /d) / DMI /d.

### Chemical analyses

**Diets**: Fresh Egyptian clover, concentrate feed mixture, and refused Egyptian clover were analyzed (replicate for every sample”) according to the method of AOAC (1997) to measure dry matter (DM, method 930.15), crude protein (CP, method 954.01), crude fiber (CF, method 962.09), ether extract (EE, method 920.39), and ash contents (method 942.05; Table 1). Nitrogen-free extract (NFE) was estimated by the difference from the sum of the protein, fat, ash, and crude fiber content.

**Table 1:** Chemical composition of the experimental diets.

	Chemical analysis					
	DM	CP	EE	CF	NFE	Ash
Commercial concentrate feed mixture*	84.81	13.48	2.14	7.47	54.28	7.43
Egyptian clover (*Trifolium alexandrinum*) as fed	18.95	2.28	0.28	5.92	7.7	2.78
Egyptian clover on a DM basis	0	12.01	1.47	31.22	40.65	14.65

DM, dry matter; CP, crude protein; EE, ether extract; CF, crude fiber; NFE, nitrogen free extract. *Concentrates mixture composed of 30% wheat bran, 22% cottonseed meal, 33% yellow corn, 10% sunflower meal, 3% molasses, 1.5% limestone, and 0.5% salt.

**Chemical composition of milk**: The percentage of milk protein, fat, lactose, and total solid, in addition to somatic cell count (SCC, cells/ml), were analyzed using a MilkoScan (130 A/SN. Foss Electric, Hilleroed, Denmark) using three aliquots (6 mL/aliquot) for each sample. Before the testing, milk samples were heated to 40°C and homogenized by vortexing for 20 s.

**Fatty acids in milk**: The total lipid content of milk was extracted using the Folch et al. (1957) method. Briefly, 2 mL of milk sample were transferred into 15 mL screw cap tubes. Then, 6 mL of a mixture containing chloroform and methanol in a ratio of 2:1 were added to the tubes, followed by vortexing for 3 min. Next, 2 mL of deionized water was added to the tubes and vortexed for 3 min. The tubes were then centrifuged for 30 min at 5000xg. The lower phase containing the extracted lipids was transferred to clean tubes and allowed to dry at room temperature. To methylate the fatty acids, sodium methoxide (2 M) was used following the method suggested by Kramer et al. (1997). The extracted lipids dissolved in 1 mL of hexane were mixed with 200 μL of sodium methoxide and vortexed. The mixture was kept for 10 min at room temperature, and then the clear top layer was transferred to gas chromatography (GC) vials. The fatty acid methyl esters (FAME) were analyzed using GC with a mass spectrometer detector (Trac 1,300, Thermo Fisher Scientific, Waltham, United States) and a TG-5MS Zebron capillary column. The FAME compounds were identified using AMDIS software^1^ based on their retention times matching the NIST library database.

**Rumen fermentation**: For volatile fatty acid (VFA) and ammonia analysis, 1 mL of rumen fluid samples were transferred into 1.5 mL Eppendorf tubes. The samples were then acidified by 200 μL of meta-phosphoric acid 25% (w/v) and stored at −20°C for later analysis. Upon thawing, samples were centrifuged at 30,000 × g (15,000 rpm, JA-17 rotor) for 20 min, and then the supernatant was used for VFA and ammonia determination. A 750 μL of supernatant was transferred to GC vials for VFA analysis by injecting 1 μL into gas chromatography (TRACE 1300, Thermo Fisher Scientific, Waltham, United States) using a capillary column (TR-FFAP 30 m × 0.53 mmI D × 0.5 μm). A standard with known concentrations of VFA was used for calibration. The other 250 μL of supernatant was used for ammonia determination colorimetrically using an ammonia assay kit (Biodiagnostic Company, Dokki, Giza, Egypt). The predicted methane was determined using the following equation: Methane yield = 316/propionate +4.4, according to Williams et al. (2019).

### Rumen microbial community

**DNA extraction and PCR amplification**: Total microbial DNA was extracted from 500 μL of rumen sample. Briefly, the sample was centrifuged at 13,000 rpm. According to the manufacturer’s instructions, DNA was extracted from the precipitated solid material using a QIAamp DNA Stool Mini Kit (Qiagen, Hilden, Germany). The quantity and quality of DNA were assessed using agarose gel electrophoresis and a Nanodrop spectrophotometer 2000 (Thermo Scientific, Massachusetts, United States). The V4 region of the 16S rRNA gene was amplified with the primers 515F and 926R using the following PCR conditions: 94°C for 3 min; 35 cycles of 94°C for 45 s, 50°C for 60 s, and 72°C for 90 s; and a final extension at 72°C for 10 min. The rumen archaeal community was studied using primers Ar915aF and Ar1386R, and the PCR amplification was conducted under the following conditions: 95°C for 5 min; 30 cycles of 95°C for 20 s, 55°C for 15 s, 72°C for 5 min, and a final extension at 72°C for 10 min. PCR amplicons were purified and sequenced using the Illumina MiSeq system.

### Bioinformatics and statistical analyses

The generated paired-end sequence reads were analyzed using the DADA2 pipeline in the R platform (Callahan et al., 2016). The fastq files of sequence reads were demultiplexed, and their quality was evaluated. Then, the sequences were filtered, trimmed, and dereplicated, followed by merging read 1 and read 2 together to get denoised sequences. Generate denoised amplicon sequence variants (ASVs), and chimeric ASVs were then removed. Taxonomic assignment of ASVs was performed using the “assign taxonomy” and “addSpecies” functions, as well as the SILVA reference database (version 138). Alpha diversity metrics were measured, including observed ASVs, Chao1, Shannon diversity, and inverse Simpson diversity indices.

Additionally, the beta diversity of the bacterial and archaeal communities was determined as principal coordinate analysis (PCoA) using Bray–Curtis dissimilarity, and figures were created using the phyloseq and ggplot R packages. The differences in alpha diversity indices, relative abundances of bacterial and archaeal phyla and dominant bacterial and archaeal genera, feed intake, rumen fermentation parameters, milk yield and composition, and FE were examined using an unpaired t-test at *p* < 0.05. Function prediction of microbial communities associated with Shami (SH) and Zaraibi (ZA) goat breeds was conducted based on the 16S rRNA data using PICRUSt2 (Douglas et al., 2020) based on the Kyoto Encyclopedia of Genes and Genomes (KEGG) database. The false discovery rate (FDR) method was used for multiple-comparison correction, and *p*-values below 0.05 were considered significant.

### Blood sampling and genotyping

For animal genotyping, blood samples were collected using vacutainer tubes containing EDTA from each animal’s jugular vein. Samples were directly transferred in an icebox to the Molecular Genetics laboratory at DRC for DNA extraction. Genomic DNA was extracted using a Puregen Core Genomic DNA extraction from blood (Qiagen^®^, Germany) according to the manufacturer’s protocol. The quantity and quality of extracted DNA were assessed using a Nanodrop spectrophotometer 2000 (Thermo Scientific, Massachusetts, United States). High-quality DNA samples (≥ 50 ng/μL) were used for genotyping at the Research Institute for Farm Animal Biology, Dummerstorf, Germany, using the Illumina®Inc. Goat_IGGC_65K_v2 Infinium HD array (Illumina, San Diego, CA, United States). SNP locations reported in this paper are based on the latest goat genome version of *Capra hircus* available from the National Center for Biotechnology Information (NCBI) database (ARS1, NCBI). The genotyping BeadChip contained 59,727 SNPs, evenly distributed throughout the entire genome. Genotype calling was performed using GenomeStudio software (Illumina *Inc*., San Diego, CA, United States) according to the manufacturer’s protocols. The quality control of genotyped SNPs was performed using PLINK v1.9 software (Chang et al., 2015), with the following filtering criteria: (i) SNPs showing significant deviation from Hardy–Weinberg Equilibrium (HWE) were excluded (*p* < 10^−6^); (ii) SNPs with a minor allele frequency (MAF) ≤ 0.01 were removed; (iii) markers with a genotype call rate < 99% and individuals with a call rate < 90% were filtered out. Additionally, SNPs mapped to unknown chromosomal positions or duplicate positions on the same chromosome were excluded to ensure data integrity for subsequent analyses.

### Genome-wide association analysis

**Genetic variance explained by markers**. The single-step GBLUP implemented in the BLUPF90 family (Legarra et al., 2014) was used to estimate the SNP effects from genomic estimated breeding values (GEBVs) of genotyped animals using the postGSf90 software of the BLUPF90 package (Aguilar et al., 2010). SNP effects were calculated as: û = DZ’ [ZDZ’]^−1^ â_g_, where û is the vector of the SNP effect; D is the diagonal matrix for weighting factors of the SNP effect; Z is the matrix of genotypes, and â_g_ is the vector of breeding values predicted for genotyped animals. The variance explained by each SNP was calculated as σ^2^ = û^2^ 2p(1 − p), where û is the SNP effect described above, and p is the allele frequency of the SNP (Zhang et al., 2010). The percentage of genetic variance explained by a window segment of 5 adjacent SNPs was calculated as: (Var (a_i_)) / (σ^2^_a_) x100%, where a_i_ is the genetic value of the *i*-th region that consists of 5 adjacent SNPs, and σ^2^_a_ is the total genetic variance (Wang et al., 2014). Manhattan plots of minus log_10_ of SNP *p*-values versus chromosomal location were drawn using the qqman package in R (Turner, 2018).

### Functional annotation, candidate genes, and gene enrichment analysis

For genome windows above the threshold (i.e., explained >0.1% of the total genetic variance), functional annotation of genes was obtained from BioMart at the Ensembl Genome Browser^2^ (Kinsella et al., 2011). Gene functions and protein domains were identified by the UniProt OMIA (Online Mendelian Inheritance in Animals) and the GeneCards databases (Safran et al., 2021). The genes that overlapped with the identified genomic interval of the candidate genome were considered candidate genes for FE in goats and were enriched using ShinyGO *v.* 0.77 (Ge et al., 2020) software. The analysis was based on gene ontology (GO; Ashburner et al., 2000) and the Kyoto Encyclopedia of Genes and Genomes (KEGG) pathways (Kanehisa et al., 2023) against the goat gene set ontologies. The program’s default parameters were applied, and the results were adjusted to a false discovery rate (FDR < 0.05).

## Results

**Feed intake**. Animals in this study were fed the same diet, which consisted of a concentrate feed mixture and fresh Egyptian clover. ZA goats exhibited significantly lower body weight compared to the SH goats (*p* < 0.05; Table 2). Goat breed significantly affected feed intake (g/kg^0.75, metabolic body weight), with ZA goats consuming more DM, CP, CF, EE, and NFE than the SH goats (*p* < 0.05).

**Table 2:** Feed intake (g/kg ^0.75^), milk yield and composition, and FE parameters in SH and ZA goat breeds.

	SH		ZA		*p*-value
	Mean	SE	Mean	SE	
Feed intake, g/kg ^0.75^					
Weight, Kg	40.6	1.39	25.3	1.57	0.0001
Dry matter intake (DMI)	110.30	1.32	136.13	3.26	0.0001
Crude protein intake (CPI)	15.35	0.13	18.84	0.45	0.0001
Ether extract intake (EEI)	2.22	0.014	2.50	0.07	0.005
Crude fiber intake (CFI)	22.89	0.46	29.34	0.97	0.0001
Nitrogen free extract (NFEI)	54.00	0.39	65.50	1.62	0.0001
Milk yield and chemical composition					
Daily milk yield (DMY), ml/day	1241	103.18	1427.77	106.18	0.22
Milk yield 3.5%, ml/day	1063.63	97.15	1301.46	129.18	0.15
Fat %	2.62	0.22	2.52	0.23	0.77
Protein %	2.29	0.058	2.55	0.07	0.016
Lactose %	4.16	0.11	4.24	0.039	0.5
Total solids (TS)%	9.71	0.19	10.00	0.28	0.42
Solids not fat (SNF)%	7.27	0.13	7.53	0.082	0.11
Somatic cell count (SCC), cells/ml	400	8.07	399.33	10.09	0.95
FE					
Gross FE	0.69	0.049	0.96	0.077	0.011
Adjusted FE	0.59	0.048	0.87	0.09	0.015
Milk net energy	0.66	0.063	0.83	0.08	0.114
FE for lactation	0.37	0.031	0.56	0.058	0.01

SE, standard error; deed efficiency = Kg milk per one Kg DM.

**Daily milk yield (DMY), chemical composition, and FE**. The estimated average DMY was higher in the ZA goats than in the SH goats. However, the difference was not significant (*p* > 0.05), and 3.5% of fat-corrected milk followed the same trend (Table 2). Furthermore, the percentages of milk fat, lactose, total solids (TS), solids not fat (SNF), and somatic cell count (SCC) did not exhibit significant differences between the studied goat breeds, but milk protein was higher in the ZA goats (2.55%) than in the SH goats (2.29%; *p* < 0.05). Based on milk yield and DMI, ZA goats exhibited greater FE expressed as gross FE and adjusted FE (Table 2; *p* < 0.05). Moreover, milk net energy was higher in the ZA goats without significant difference, and FE for lactation was higher in the ZA goats compared to the SH goats (*p* < 0.05; Table 2).

**Milk fatty acids:** The most abundant fatty acids identified were palmitic (C16:0), oleic (C18:1-cis), stearic (C18:0), myristic (C14:0), capric (C10:0), lauric (C12:0), and linoleic (C18:2-cis-9.12; Table 3). The proportions of certain fatty acids in milk were influenced by the breed of goat. The SH goats exhibited higher percentages of caproic (C6:0), myristic (C14:0), palmitic (C16:0), and total saturated fatty acids (∑ SFA; *p* < 0.05). In contrast, the ZA goats exhibited higher percentages of tridecylic (C13:0), heptadecanoic (C17:0), stearic (C18:0), behenic (C22:0), pentadecenoic (C15:1, cis-10), oleic (C18:1, cis-9), elaidic (C18:1, trans-9), rumelenic (C18:2, cis 9, trans 11), mono-unsaturated fatty acids (∑ MUFA), and polyunsaturated fatty acids (∑ PUFA; *p* < 0.05; Table 3).

**Table 3:** Milk fatty acids (%) in Shami (SH) and Zaraibi (ZA) goat breeds.

	SH		ZA		*p*-value
	Mean	SE	Mean	SE	
C6:0	1.89	0.17	1.20	0.21	0.042
C8:0	2.45	0.13	1.82	0.27	0.08
C10:0	9.01	1.29	6.11	0.74	0.09
C12:0	4.85	0.27	4.45	0.33	0.38
C13:0	0.17	0.00	0.88	0.01	0.0001
C14:0	12.38	0.39	9.64	0.13	0.001
C15:0	0.95	0.09	1.12	0.10	0.24
C16:0	34.60	0.30	23.68	0.71	0.0001
C17:0	0.65	0.06	1.13	0.15	0.027
C18:0	8.93	1.23	15.98	1.65	0.014
C20:0	0.66	0.14	1.11	0.36	0.30
C21:0	0.41	0.11	0.79	0.21	0.15
C22:0	0.53	0.096	1.05	0.18	0.049
C15:1, cis-10	0.25	0.01	0.48	0.03	0.0001
C16:1, trans-9	0.61	0.17	1.09	0.20	0.12
C17:1, cis-10	0.48	0.10	0.58	0.09	0.51
C18:1, cis-9	14.50	0.68	18.71	0.96	0.01
C18:1, trans-9	2.82	0.39	5.53	0.21	0.001
C18:1, cis-13	0.77	0.012	0.73	0.019	0.21
C18:2, cis-9,12	2.3	0.12	1.83	0.31	0.21
C18:2, cis- 9, trans- 11	0.45	0.012	1.26	0.07	0.0001
C20:5	0.29	0.026	0.81	0.26	0.09
∑ SFA	56.12	1.25	53.21	0.48	0.07
∑ MUFA	19.44	0.86	27.12	0.90	0.001
∑ PUFA	3.05	0.14	3.91	0.6	0.20

SFA, saturated fatty acid; MUFA, monounsaturated fatty acid; PUFA, polyunsaturated fatty acid; SE, standard error.

**Rumen fermentation**: The concentrations of acetic, propionic, and total VFA were higher in the ZA goats (*p* < 0.05; Table 4). Additionally, rumen ammonia, isovaleric, and predicted methane were higher in the SH goats (*p* < 0.05; Table 4).

**Table 4:** Rumen fermentation parameters in Shami (SH) and Zaraibi (ZA) goat breeds.

	SH		ZA		*p*-value
	Mean	SE	Mean	SE	
PH	6.42	0.07	6.52	0.06	0.29
Acetic, mM	40.50	2.63	47.73	0.75	0.017
Propionic, mM	13.41	1.32	17.49	1.10	0.029
Isobutyric, mM	1.31	0.18	0.76	0.24	0.09
Butyric, mM	13.92	1.03	13.72	0.73	0.87
Isovaleric, mM	1.54	0.27	0.73	0.08	0.008
Valeric, mM	2.13	0.18	2.30	0.17	0.51
Total VFA, mM	72.83	4.95	82.76	1.82	0.07
Ammonia/ mmol/L	37.10	4.40	24.51	1.57	0.015
Predicted methane, g/kg DMI	30.23	3.11	22.69	1.12	0.035

SE, standard error; DMI, dry matter intake; VFA, volatile fatty acids.

### Microbial community

**Figure 1:**
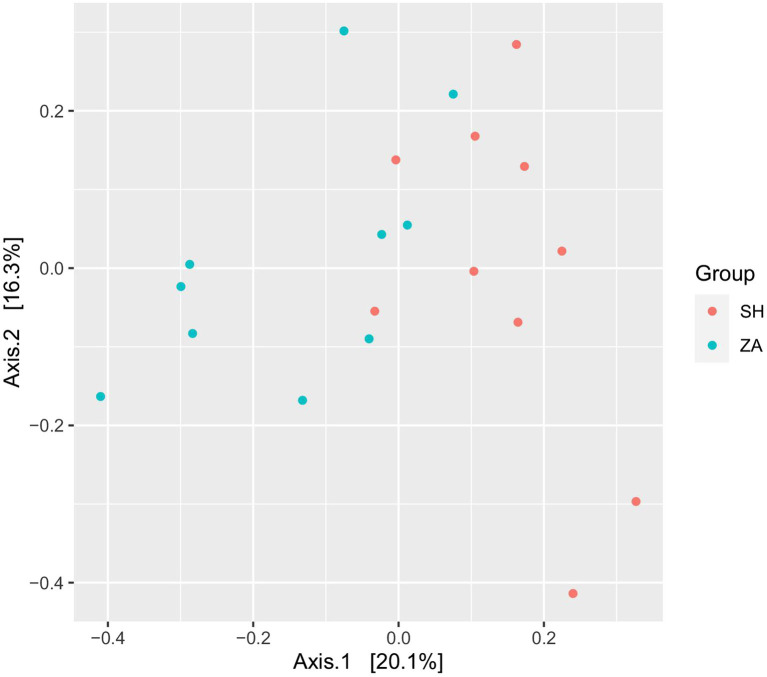
Principal coordinates analysis (PCoA) of bacterial community. PCoA of rumen bacteria in lactating goats based on Bray-Curtis dissimilarity. The analysis was conducted between two goats’ breeds: red circles for Shami breed (SH), blue circles for Zaraibi breed (ZA).

**Diversity of bacterial community**: The total high-quality non-chimeric reads in the SH and ZA goats were 570,677 and 666,870, respectively, and the difference in the average reads number between the goat groups was not significant (*p* > 0.05; Table 5). Alpha diversity was calculated as observed ASVs, Chao1, Shannon, and inverse Simpson. (Table 5) showed that the ZA goat group had numerically lower alpha diversity metrics without significant differences (*p* > 0.05). Principal coordinate analysis (PCoA) based on the Bray-Curtis distance was conducted to show the similarity between microbial communities (Figure 1). The results revealed that bacterial communities were clustered separately based on the goat breeds.

**Table 5:** Alpha diversity indexes of rumen bacteria and relative abundances (%) of bacterial phyla in the rumen of SH and ZA goat breeds.

	SH		ZA		*p*-value
	Mean	SE	Mean	SE	
Observed	518.5	35.55	514.9	30.66	0.94
Chao1	518.5	35.55	514.9	30.66	0.94
Shannon	4.51	0.16	4.49	0.088	0.90
InviSimpson.	35.93	7.01	30.05	3.36	0.46
Bacterial phyla (%)					
Bacteroidota	85.06	1.59	87.54	1.13	0.21
Cyanobacteria	0.15	0.044	0.09	0.033	0.26
Desulfobacterota	0.039	0.007	0.06	0.018	0.31
Fibrobacterota	0.24	0.035	0.5	0.06	0.002
Firmicutes	14.77	1.014	11.03	1.10	0.023
Planctomycetota	0.067	0.015	0.05	0.008	0.43
Spirochaetota	0.45	0.07	0.60	0.08	0.18
Synergistota	0.53	0.17	0	0	0
Verrucomicrobiota	0.10	0.013	0.07	0.014	0.11

SE, standard error.

**Structure of bacterial community**: At the phylum level, the bacterial community in the rumen of goats was affiliated with eight bacterial phyla, including Bacteroidota, Cyanobacteria, Desulfobacterota, Fibrobacterota, Firmicutes, Planctomycetota, Spirochaetota, and Verrucomicrobiota, which were shared between all rumen samples (Table 5). In addition, one bacterial phylum, Synergistota, was observed only in the SH goats. The goat breed affected the relative abundance of bacterial groups (Table 5). Bacteroidota and Firmicutes were the most abundant bacterial phyla, representing more than 98% of the bacterial community. The Bacteroidota phylum dominated the bacterial community and accounted for 85.06 and 87.54% in the SH and ZA goats, respectively. This phylum was classified mainly into the families Prevotellaceae, Rikenellaceae, Bacteroidales RF16 group, Bacteroidales p-251-o5, Bacteroidales F082, Muribaculaceae, and Bacteroidales BS11 gut group (Table 6). Except for the family Prevotellaceae, other families showed higher relative abundance in the SH goats. Family Prevotellaceae represented the most abundant family and accounted for 60.38% in the SH goats and 66.57% in the ZA goats with a significant difference (*p* < 0.05); this family was classified mainly into two genera, *Prevotella* and *Prevotellaceae NK3B31 group,* that were higher in the ZA goats. Family Rikenellaceae was dominated by the *Rikenellaceae RC9 gut group,* which was higher in the SH goats (Table 6).

**Table 6:** Relative abundances (%) of dominant bacterial families (F) and genera (G) in the rumen of Shami (SH) and Zaraibi (ZA) goat breeds.

		SH		ZA		*p*-value
		Mean	SE	Mean	SE	
Phylum: Bacteroidota						
F: Prevotellaceae		60.38	3.20	66.57	2.24	0.13
G: Prevotella		50.06	4.31	60.39	2.21	0.047
G: Prevotellaceae NK3B31 group		2.08	1.13	0.96	0.16	0.35
F: Rikenellaceae		4.70	0.48	4.39	0.30	0.59
G: Rikenellaceae RC9 gut group		4.46	0.45	4.25	0.29	0.70
F: Bacteroidales RF16 group		3.13	0.72	2.55	0.42	0.50
F: Bacteroidales p-251-o5		2.85	0.94	0.81	0.26	0.06
F: Bacteroidales F082		8.53	0.63	7.76	0.77	0.45
F: Muribaculaceae		4.78	0.51	4.59	0.47	0.78
F: Bacteroidales BS11 gut group		0.18	0.053	0.13	0.047	0.55
Phylum: Firmicutes						
F: Ruminococcaceae		2.20	0.44	1.47	0.50	0.29
F: Oscillospiraceae		2.28	0.42	1.51	0.25	0.13
F: Oscillospiraceae	G: NK4A214 group	1.77	0.35	1.11	0.239	0.13
F: Hungateiclostridiaceae	G: Saccharofermentans	0.25	0.03	0.30	0.06	0.43
F: Christensenellaceae		2.02	0.37	1.01	0.21	0.03
F: Christensenellaceae	G: Christensenellaceae R-7 group	1.97	0.37	0.98	0.21	0.03
F: Lachnospiraceae		1.83	0.23	1.83	0.13	0.98
F: Lachnospiraceae	G: Lachnospiraceae AC2044 group	0.06	0.008	0.14	0.021	0.003
F: Lachnospiraceae	G: Butyrivibrio	0.39	0.07	0.35	0.04	0.61
F: Lachnospiraceae	G: Lachnospiraceae NK3A20 group	0.14	0.015	0.21	0.024	0.03
F: Lachnospiraceae	G: Acetitomaculum	0.08	0.02	0.05	0.008	0.20
F: Defluviitaleaceae	G: Defluviitaleaceae UCG-011	0.039	0.008	0.027	0.004	0.25
F: Acidaminococcaceae	G: Succiniclasticum	0.05	0.013	0.23	0.04	0.001
F: Acholeplasmataceae	G: Anaeroplasma	0.199	0.024	0.30	0.05	0.09
F: Anaerovoracaceae	G: Family XIII AD3011 group	0.05	0.01	0.02	0.005	0.06
F: Anaerovoracaceae	G: Anaerovorax	0.06	0.011	0.05	0.010	0.61
Phylum: Spirochaetota						
F: Anaerovoracaceae	G: Mogibacterium	0.04	0.010	0.02	0.005	0.049
F: Spirochaetaceae	G: Treponema	0.28	0.05	0.36	0.05	0.28
F: Spirochaetaceae	G: Sphaerochaeta	0.10	0.03	0.07	0.015	0.49

SE, standard error.

Phylum Firmicutes accounted for 14.77% of the SH goats and 11.03% of the ZA goats, with a significant difference (*p* < 0.05; Table 5). This phylum was classified mainly into four families, Ruminococcaceae, Oscillospiraceae, Christensenellaceae, and Lachnospiraceae, which were higher in the SH goats (*p* < 0.05; Table 6). Family Oscillospiraceae was affiliated mainly with the genus *NK4A214 group*, family Christensenellaceae was affiliated mainly with the genus *Christensenellaceae R-7 group*, and family Lachnospiraceae was affiliated mainly with the *Lachnospiraceae NK3A20 group* that was higher in the ZA goats (*p* < 0.05) in addition to the genus *Butyrivibrio* that was higher in the SH goats (Table 6). Phylum Fibrobacterota and Spirochaetota were more abundant in the ZA goats compared to the SH goats (*p* < 0.05; Table 5).

**Figure 2:**
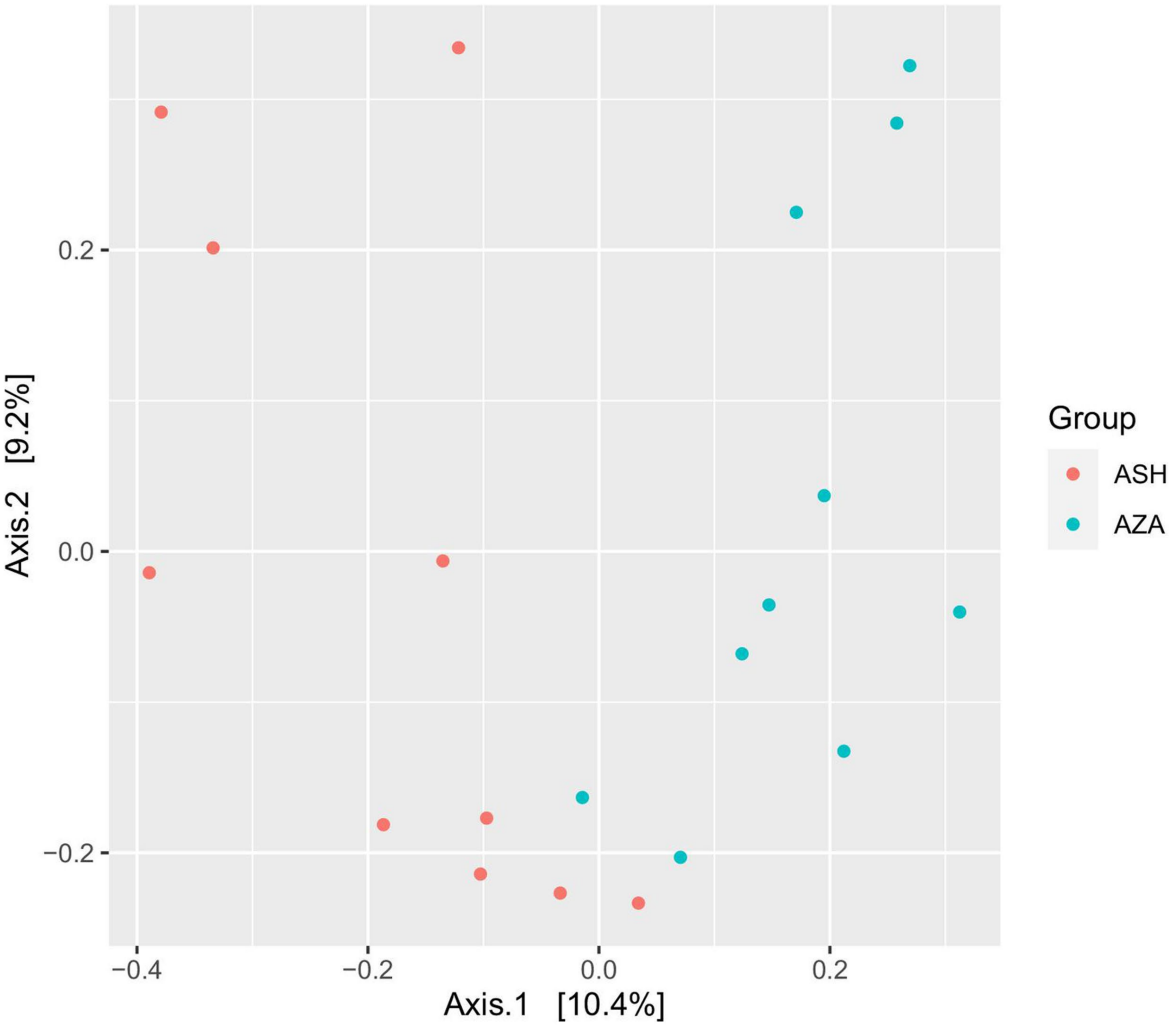
Principal coordinates analysis (PCoA) of archaeal community. The analysis was conducted between two goat breeds: red circles for the Shami breed (ASH) and blue circles for the Zaraibi breed (ZA).

**Diversity of archaeal community**: After data processing and the chimeras were removed, a total of 40,313 remained in group SH, and 61,061 reads remained in the ZA goats without significant difference (*p* > 0.05; Table 7). Alpha diversity, including observed ASVs, Chao1, Shannon, and Invisimpsone, was similar between the goat breeds, and the ZA goats showed numerically higher observed ASVs, Chao1, and Shannon, and the SH goats exhibited higher Invisimpsone (Table 7; *p* > 0.05). Principal coordinate analysis (PCoA) based on the Bray-Curtis of archaeal community across the goat breeds demonstrated that the samples were clustered based on the animal breeds (Figure 2).

**Table 7:** Alpha diversity indexes of rumen archaea and relative abundances (%) of archaeal genera in the rumen of Shami (SH) and Zaraibi (ZA) goat breeds.

	SH		ZA		*p* value
	Mean	SE	Mean	SE	
Diversity of archaeal community					
Observed	36.4	4.62	43.4	4.92	0.31
Chao1	36.4	4.62	43.4	4.92	0.31
Shannon	2.76	0.21	2.80	0.14	0.86
InviSimpson.	12.04	1.90	11.06	1.64	0.70
Relative abundance of archaeal genera					
P: Thermoplasmatota, F: Methanomethylophilaceae,					
Candidatus Methanomethylophilus	18.45	5.35	64.60	7.94	0.0001
Methanomethylophilaceae_unclass	20.20	5.58	15.67	2.03	0.45
P: Euryarchaeota, F: Methanobacteriaceae,					
Methanobrevibacter	61.44	8.13	23.04	7.10	0.002
Methanosphaera	0.98	0.154	0.62	0.18	0.15

P, Phylum; F, family; SE, standard error.

**Structure of archaeal community**: The taxonomic analysis of the archaeal community revealed that the ASVs were assigned to two phyla, Thermoplasmatota, which was further classified to the family Methanomethylophilaceae, and phylum Euryarchaeota was affiliated with the family Methanobacteriaceae (Table 7). Goat breed affected the archaeal community, and the family Methanomethylophilaceae had two genera, *Candidatus Methanomethylophilus,* that accounted for 18.45% in group Shami goats (ASH) and 64.60% in Zaraibi goats (AZA) group with a significant difference (*p* < 0.05), and *unclassified Methanomethylophilaceae* that was higher in the SH goats compared to AZA. The family *Methanobacteriaceae* was further categorized into two genera. *Methanobrevibacter* constituted 61.44% of the archaeal community in the SH goats and 23.04% in the AZA group, showing a significant difference (p < 0.05). Additionally, the genus *Methanosphaera* was more prevalent in the SH goats compared to AZA (Table 7).

**Figure 3:**
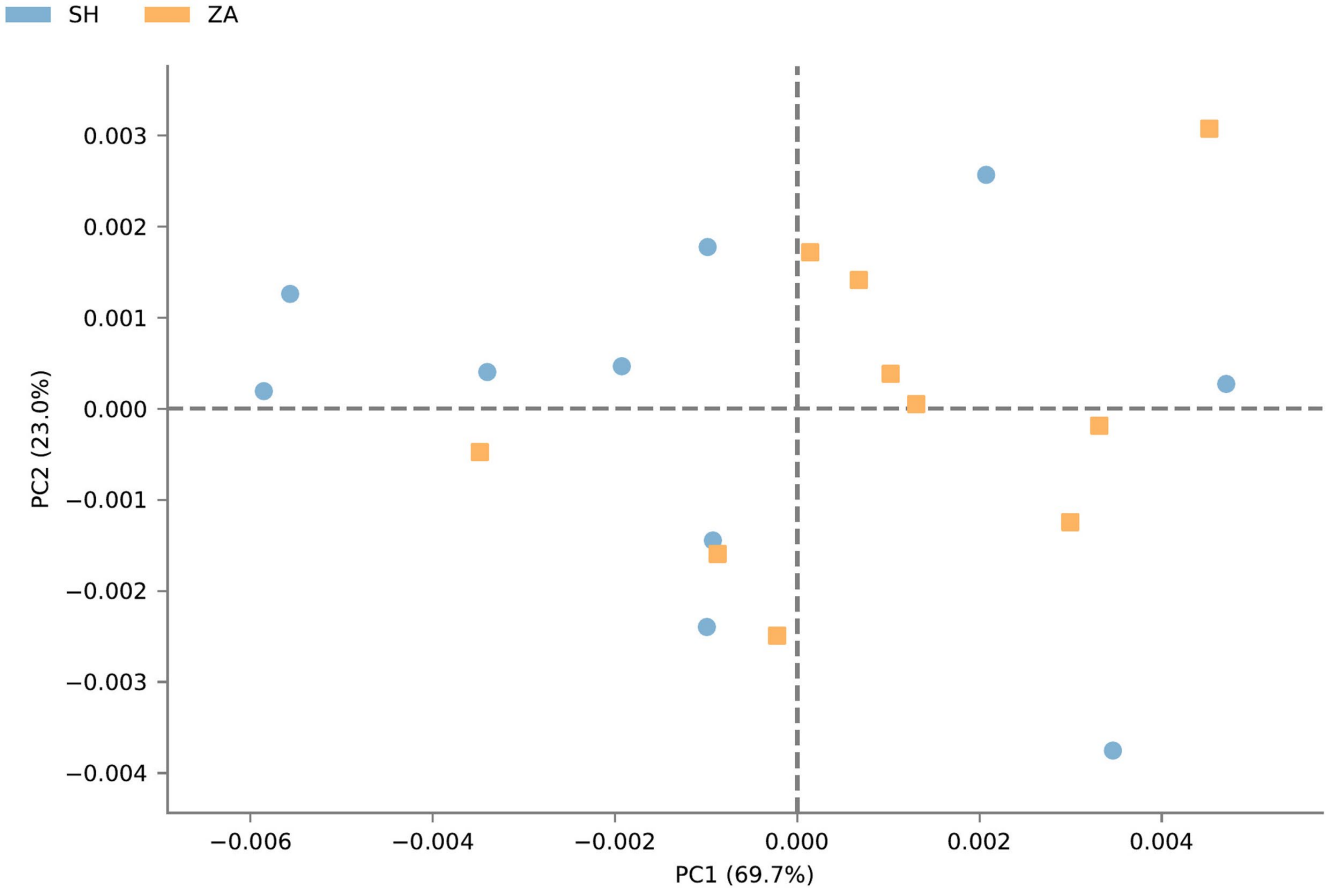
Principal components analysis of PICRUSt2 functional prediction of microbial communities in the rumen of Shami and Zaraibi goat breeds. Orange squares refer to ZA samples, and blue circles refer to SH’s samples.

**Figure 4:**
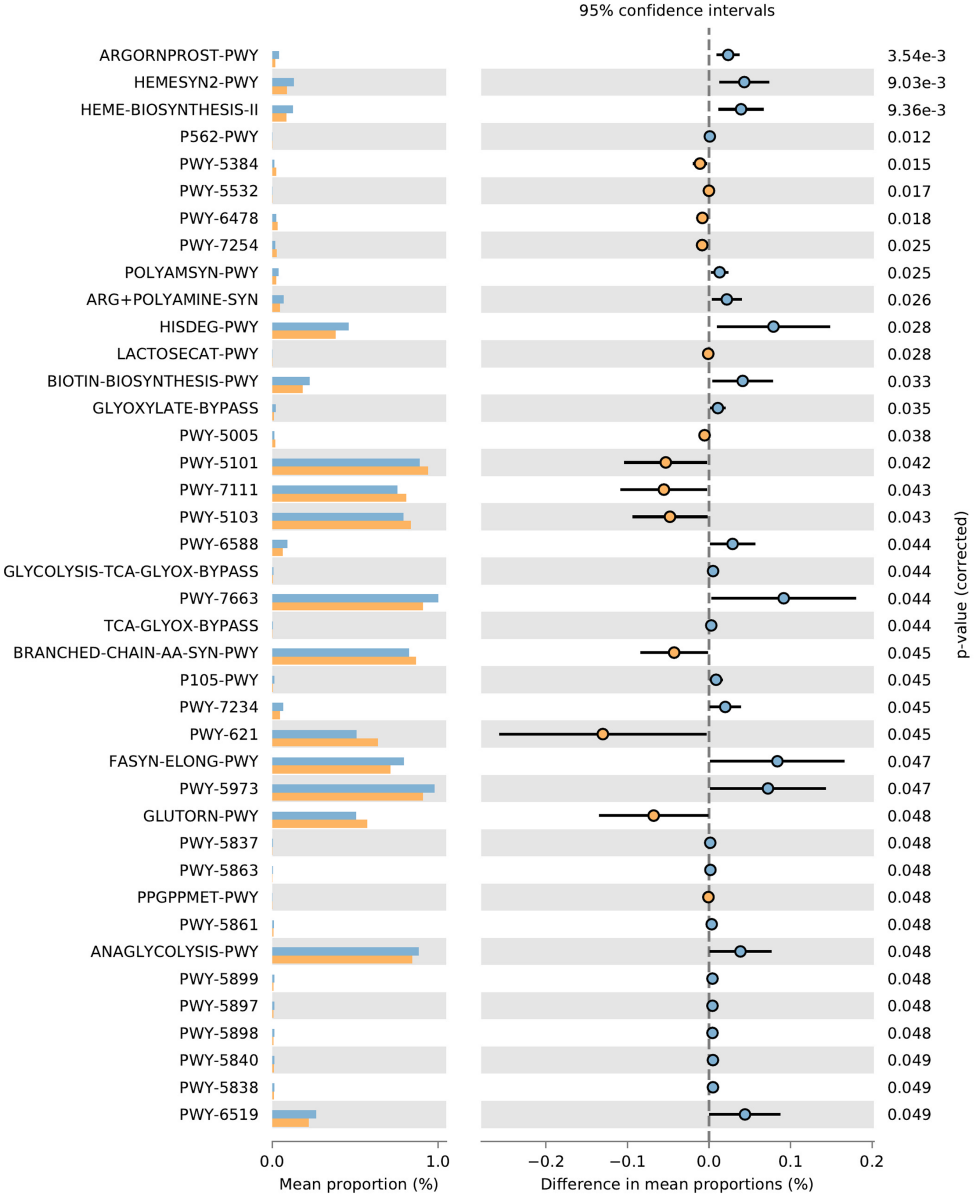
Effect of animal species on the relative abundances of metabolic pathways of rumen microbial communities in the rumen of SH and ZA goat breeds. Orange pars refer to ZA’s samples, and blue pars refer to SH’s samples.

**Function prediction of microbial community**: The metabolic pathways of rumen microbial communities in Shami (SH) and Zaraibi (ZA) goats were performed using PICRUSt2. Principal components analysis (PCA) of PICRUSt2 function prediction was generated using the relative abundance of metabolic pathways (Figure 3), which revealed that samples were clustered based on animal breeds. The samples of the SH goats revealed significantly higher (q < 0.05) relative abundances of metabolic pathways related to heme biosynthesis-II, L-arginine degradation (ARGORNPROST-PWY), P562-PWY, polyamine biosynthesis I (POLYAMSYN-PWY), ARG + POLYAMINE-SYN, L-histidine degradation I (HISDEG-PWY), biotin biosynthesis-PWY, the glyoxylate cycle (GLYOXYLATE-BYPASS), pyruvate fermentation to acetone (PWY-6588), the glyoxylate cycle and TCA cycle (GLYCOLYSIS-TCA-GLYOX-BYPASS), anaerobic gondoate biosynthesis (PWY-7663), coenzyme B biosynthesis (TCA-GLYOX-BYPASS), and the tricarboxylic acid (TCA) cycle IV (2-oxoglutarate decarboxylase; P105-PWY; Figure 4). Additionally, the samples of group ZA revealed significantly higher (q < 0.05) relative abundances of metabolic pathways related to ascorbate and aldarate metabolism, carbohydrate metabolism, nitrogen metabolism, sulfur metabolism, lactose and galactose degradation (LACTOSECAT-PWY), amino acid metabolism (BRANCHED-CHAIN-AA-SYN-PWY), L-ornithine biosynthesis I (GLUTORN-PWY), glycolysis III (ANAGLYCOLYSIS-PWY), the sucrose degradation pathway (PWY-5384), nucleoside and nucleotide degradation (PWY-5532), GDP-d-glycero-alpha-d-manno-heptose biosynthesis (PWY-6478), and the tricarboxylic acid cycle citric acid cycle (PWY-7254; Figure 4).

**Figure 5:**
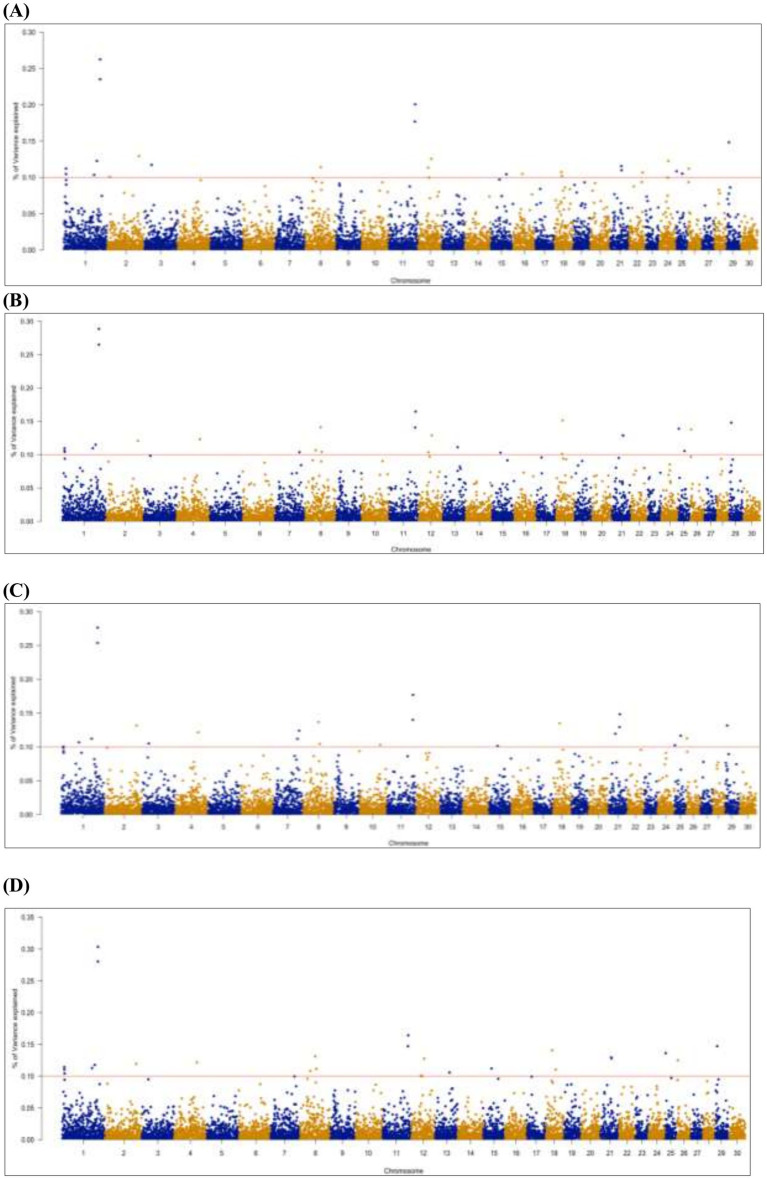
Manhattan plots of genome-wide association results for gross FE **(A)**, adjusted FE **(B)**, milk net efficiency **(C),** and FE for lactation **(D)** in Egyptian goats. Each dot represents a genome window. The scale of the y-axis represents the significance of the percentage of genetic variance explained by the genome window, and chromosomes are displayed on the x-axis.

**Genetic variance explained by markers**: A significant genome window was considered to contribute to the genetic variance of FE traits if it accounted for more than 0.1%. A total of 26 genome windows, each explaining >0.1% of the total genetic variance explained by all windows, with a total of 2.32% of genetic variance (Figure 5). These windows were located on chromosomes 1 (6, 110, and 132 Mb), 2 (9 and 116 Mb), 3 (24 Mb), 7 (88 and 94 Mb), 8 (60 Mb), 10 (76 Mb), 11 (94 and 95 Mb), 12 (47 Mb), 13 (53 Mb), 15 (31 Mb), 18 (21, 23, 25, and 36 Mb), 21 (25 and 41 Mb), 22 (49 Mb), 24 (33 Mb), 25 (1 and 22 Mb), and 26 (3 Mb; Table 8; Supplementary file 1).

**Table 8:** Percentages of variance explained by genome windows and the annotated genes for FE traits in goats.

CHI^1^	Window start	Window end	Annotated genes	Trait (% variance explained)^2^
1	132,018,470	132,277,390	*PCCB, SLC35G2, PPP2R3A*	G (0.26), AFE (0.28), MNE (0.27), FEL (0.3)
1	110,599,971	110,922,640	*KCNAB1*	G (0.1), AFE (0.11), MNE (0.11), FEL (0.11)
1	64,366,087	64,625,165	*PLA1A, GSK3B*	MNE (0.11)
2	116,526,246	116,676,692	*MTX2, AGPS*	G (0.13), AFE (0.12), MNE (0.13), FEL (0.12)
2	9,509,950	9,686,517	*CD52, CRYBG2, PIGV, WDTC1*	G (0.1), MNE (0.1)
3	24,944,827	25,112,029	*–*	MNE (0.1)
7	88,275,396	88,422,191	*FNIP1, ACSL6, SLC22A4, and SLC22A5*	AFE (0.10), MNE (0.11)
7	94,439,034	94,608,030	*–*	MNE (0.12)
8	60,125,954	60,391,464	*GBA2*	AFE (0.1), MNE (0.1), FEL (0.11)
10	76,919,396	77,109,418	*RAB2B*	MNE (0.1)
11	95,676,545	95,956,303	*GOLGA1, RABEPK, GAPVD1*	G (0.2), AFE (0.16), MNE (0.17), FEL (0.16)
11	94,119,124	94,320,022	*RABGAP1, DENND1A*	G (0.17), AFE (0.14), MNE (0.14), FEL (0.14)
12	47,309,742	47,525,212	*COMMD6, TBC1D4*	AFE (0.12), MNE (0.12)
13	53,615,692	53,737,265	*EEF1A2, SLC17A9, SLCO4A1*	AFE (0.11), FEL (0.1)
15	31,003,291	31,254,796	*STARD10, VPS26B, ACAD8, GLB1L3*	AFE (0.11), MNE (0.1), FEL (0.11)
18	21,467,618	21,638,976	*GPT2*	AFE (0.1)
18	23,894,157	24,074,733	*IRX3, FTO*	G (0.1), AFE (0.15), MNE (0.13), FEL (0.14)
18	25,187,303	25,456,930	*LPCAT2, MT4, BBS2*	G (0.17)
18	36,786,723	36,966,167	*HSD11B2, NRN1L, LCAT, SLC12A4, DPEP3, SLC7A6*	FEL (0.11)
21	41,944,739	42,157,306	*PRKD1, SCFD1, AP4S1*	G (0.11), AFE (0.12), MNE (0.15), FEL (0.13)
21	25,663,319	25,811,086	*MTHFS*	MNE (0.12)
22	49,812,140	49,976,426	*CACNA2D2, TMEM115, HYAL2, SLC38A3*	G (0.1)
24	33,746,826	33,895,390	*TMEM241*	G (0.12)
25	1,683,490	1,907,730	*ECI1, ABCA3, TBC1D24, ATP6V0C, PDPK1*	G (0.11), AFE (0.13), MNE (0.1), FEL (0.13)
25	22,172,049	22,460,983	*SLC5A11*	G (0.1), AFE (0.1), MNE (0.11)
26	3,792,132	3,973,839	*HEATR5A, NUBPL, MVP*	G (0.12), AFE (0.13), FEL (0.12)

1 CHI, chromosome.

2 G, gross FE; AFE, adjusted FE; MNE, milk net energy; FEL, FE for lactation.

**Figure 6:**
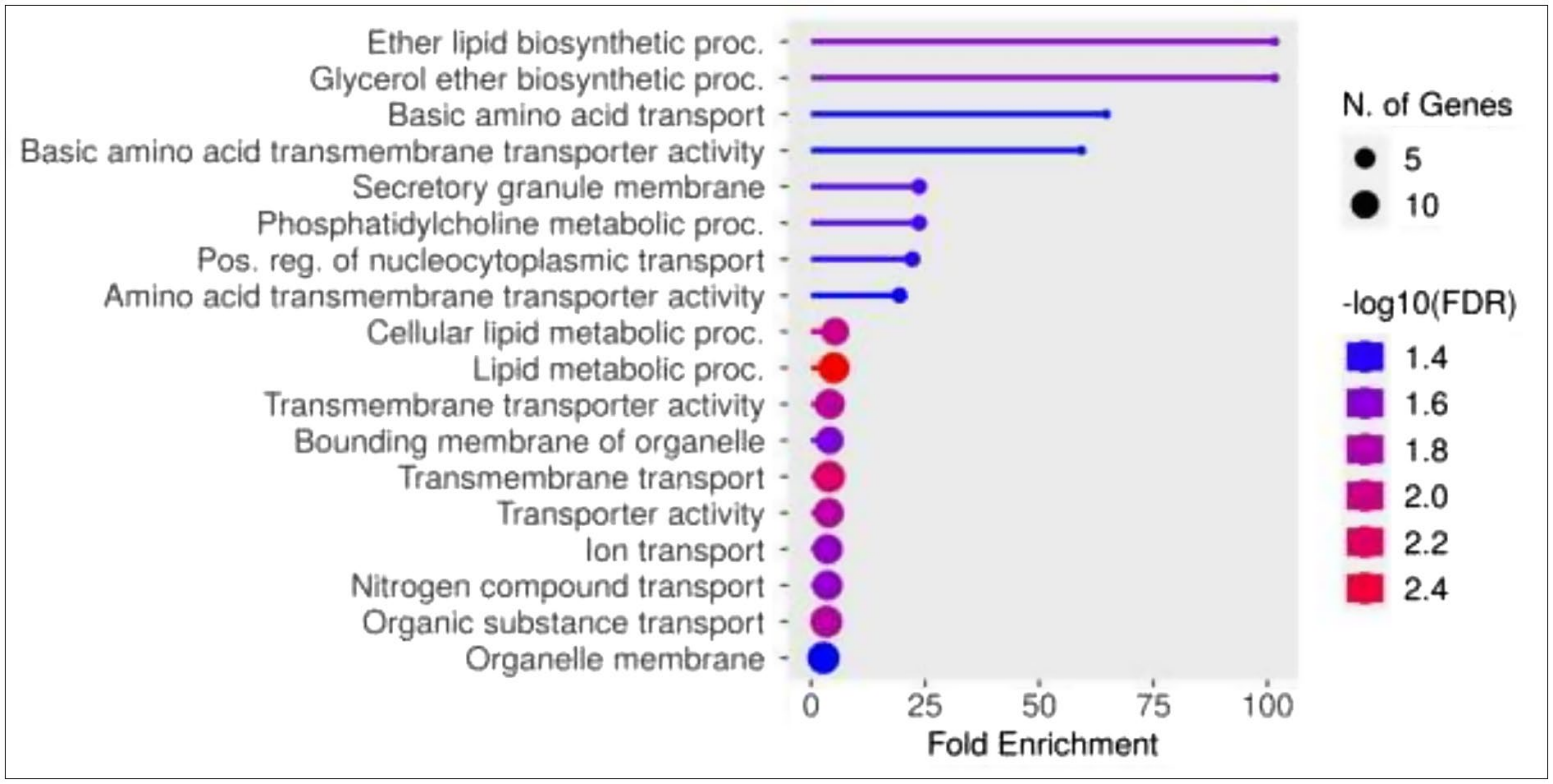
Gene ontology (GO) enrichment analysis of biological pathways for the list of candidate genes resulted from genome-wide association analysis for FE in Egyptian goats. False discovery rate (FDR) < 0.05.

**Gene enrichment analysis**: The gene enrichment analysis identified 26 significant (q < 0.05) biological pathways using a list of the identified candidate genes in the current study (Supplementary Excel file S1). The identified candidate genes were significantly enriched in biological pathways related to the regulation of the transport process of many cellular components such as ions, lipids, organic substances, amino acids, and the general transporter activities mechanism (Figure 6).

## Discussion

FE represents an animal’s capacity to convert feed components into products such as meat and milk through rumen microbial fermentation. This efficiency directly impacts the profitability of livestock production enterprises (Fischer et al., 2022). Therefore, investigating the relationship between the rumen microbiome, animal genotype, and FE is crucial for genetic improvement programs in livestock species comprising goats. In the current study, goats’ diets consisted of a mixture of feed concentrates and fresh clover, which is a high-quality roughage. Estimates of milk yield and feed intake values were in agreement with the values reported by Ghoneem and El-Tanany (2023). On the other hand, milk yield in the current study (Table 2) was higher than those obtained by Alsheikh (2013) and Kholif et al. (2020) for Shami and Zaribi goat breeds. To the best of our knowledge, no studies compared the FE, feed intake, and milk yield between the SH and ZA goats under the same husbandry conditions. However, the studies conducted by Ghoneem and El-Tanany (2023) and Kholif et al. (2020) reported estimates of FE in the SH and ZA goats similar to our study.

ZA goats achieved higher FE (0.96 kg milk/kg DM) than the SH goats (0.69; *p* < 0.05), likely due to their superior feed utilization and adaptation to the arid conditions of southern Egypt, their region of origin (Alsheikh, 2013). Similarly, Knights and Garcia (1997) reviewed Zaraibi goats as producing higher milk than Shami goats.

Variations in feed intake, milk yield, and composition (Tables 2, 3), and FE between breeds were reported in different ruminant species (Knights and Garcia, 1997; Paz et al., 2016; Currò et al., 2019; Gustavsson et al., 2014; Niero et al., 2023). Bharanidharan et al. (2018) reported that high-efficiency dairy cows showed higher feed intake and higher milk yield. Our results showed that ZA goats exhibited higher levels of milk protein and unsaturated fatty acids (∑ PUFA), which is consistent with the higher abundance of *Prevotella* observed. The breed-specific differences in milk fatty acid profiles observed in this study align with previous findings in goats and sheep (Idamokoro et al., 2019). This could be attributed to variations between animal species and breeds in metabolic pathways of lipids in mammary glands and rumen microorganisms (Conte et al., 2022). Therefore, it is necessary to understand the differences in milk fat profiles in different livestock breeds.

Zhang et al. (2023) and Sasson et al. (2017) explained that host genome and rumen microbiome impact milk protein, which could be altered due to a higher abundance of rumen bacteria: *Prevotella*, *Bacteroidales*, *Clostridiales*, *Flavefaciens*, and *Ruminococcus*. Additionally, the proportion of polyunsaturated fatty acids (PUFA) is affected mainly by rumen microorganisms through rumen biohydrogenation (Shingfield et al., 2010; Huws et al., 2011). In this regard, Hu et al. (2020) identified a positive correlation between the higher *Prevotella* and higher unsaturated fatty acids (UFA) in yaks, as *Prevotella* play a vital role in energy harvesting in the rumen, and it might provide precursors for UFA synthesis, which demonstrates the higher UFA in ZA compared to the SH goats in this study. A similar conclusion was obtained by Huws et al. (2011), who studied the bacteria that play a predominant role in ruminal biohydrogenation.

### Rumen ecosystem and FE

The rumen bacterial community in goats was dominated by the phyla Bacteroidota, Firmicutes, Spirochaetota, and Fibrobacterota (Table 5), aligning with findings from previous studies on goats (Giger-Reverdin et al., 2020; Luo et al., 2022). The Firmicutes phylum was higher in the SH goats with lower FE, which was also reported in low-efficiency beef cattle by Brooke et al. (2019).

Host genetics significantly influence the rumen microbiome, with approximately 35% of microbial taxa being heritable (Li et al., 2019). This implies that the difference in the microbial communities between the two goat breeds in the current study is expected. In this context, our PCoA analysis (Figure 1) showed that the bacterial communities in SH and ZA breeds were distinct, which agrees with the findings of Paz et al. (2016) in Holstein and Jersey breeds. Similar results were obtained by Mani et al. (2021) in the rumen of Damara and Meatmaster sheep breeds in South Africa. In the current study, ZA showed numerically low alpha bacterial diversity compared to the SH goats (*p* > 0.05; Table 5), which is similar to other findings in dairy cows (Shabat et al., 2016).

The variation in the composition of rumen microbiota results in changes in rumen fermentation efficiency, wherever the ZA goats had lower ammonia than the SH goats (24.51 vs. 37.10 mmol/L, *p* < 0.05). Furthermore, the ZA goats showed higher total VFA than the SH goats (82.76 vs. 72.83 mM, *p* < 0.05) as well as higher acetic and propionic (Table 4; *p* < 0.05). VFAs represent the main energy source of the host animal (Paz et al., 2016). Nitrogen and carbohydrate metabolism are the main microbial functions in the rumen (Xue et al., 2022). Higher VFA production in ZA was associated with higher fiber-degrading bacteria and higher carbohydrate metabolism pathways (Table 4; Figure 4), which agrees with Tapio et al. (2017) and Xue et al. (2022), indicating that Zaraibi goats have higher efficiency in carbohydrate metabolism. This finding is supported by higher relative abundances of Fibrobacters, Prevotellaceae, and Lachnospiraceae in the rumen of the ZA goats. These bacterial groups have essential roles in complex carbohydrates and protein metabolism (Thoetkiattikul et al., 2013; Paz et al., 2016; Neumann et al., 2018). Xue et al. (2022) reported that pathways related to carbohydrate metabolism could be used as prediction markers to differentiate efficient and inefficient rumen microbiomes or animals, which helps the future selection of high-efficiency animals. These findings were supported by Wang et al. (2023), who reported higher carbohydrate metabolism pathways in goats with higher growth performance. On the other hand, the ZA goats showed higher relative abundances of branched-chain amino acids (BCAA) pathways. The increase in the relative abundance of branched-chain amino acids (BCAA) pathways could refer to a higher concentration of BCAA in the rumen. Zhang H. L. et al. (2013) reported that BCAA stimulates fiber-degrading microorganisms and VFA production.

Furthermore, the increment in acetic acid production was accompanied by an increment in the PWY-7254 pathway (tricarboxylic acid cycle citric acid cycle), which is involved in the synthesis of precursors of acetic acid (De Sales-Millán et al., 2024). Group ZA showed lower rumen ammonia and higher relative abundances of pathways related to nitrogen metabolism, which could indicate the efficient use of nitrogen in microbial protein synthesis and the decline in nitrogen loss (Firkins et al., 2007). Higher synthesis of microbial protein increases the protein supply to the host animals Zhang H. L. et al. (2013). Furthermore, the ZA goats showed higher sulfur metabolism, which is linked to lower methane production (Wu et al., 2021), as sulfate-reducing bacteria are able to compete with methanogens for H_2_ in the rumen, which inhibits the methanogenesis (Zhao and Zhao, 2022).

Genus *Prevotella* is a major player in ruminal metabolism and predominated the rumen microbiome in several ruminant species (Betancur-Murillo et al., 2022). A higher relative abundance of genus *Prevotella* was associated with the highly efficient breed (i.e., ZA; Tables 2, 6). This finding is supported by Brooke et al. (2019), who reported that the *Prevotella* is a microbial marker identifying cattle with higher FE. Genus *Prevotella* includes members that utilize a wide range of substrates, such as hemicellulose and protein (Matsui et al., 2000). Myer et al. (2015) found a higher relative abundance of *Prevotella* in the gut of steers with high daily gain and intake. De Vadder et al. (2016) indicated that the genus *Prevotella* produces succinate, which is the precursor to propionate. The propionate synthesis uses hydrogen molecules, reducing its availability for methane production by methanogenic archaea (Betancur-Murillo et al., 2022). Thus, *Prevotella* has the potential to be used as an anti-methanogenic agent.

The members of the family Lachnospiraceae have fibrolytic and cellulolytic activities and were associated with higher milk production in Holstein cows (Thoetkiattikul et al., 2013; Paz et al., 2016). In this study, the candidate genus Lachnospiraceae NK3A20 was higher in the ZA goats; this genus was reported to produce butyric acid that promotes the development of the rumen (Huang et al., 2021). Boeckaert et al. (2009) indicated that the Lachnospiraceae NK3A20 group plays an important role in the efficiency of energy utilization. Genus *Succiniclasticum* is one of the dominant bacteria in the rumen of heifers (Liu et al., 2017), and it converts the succinate to propionate (van Gylswyk, 1995). Shabat et al. (2016) explained that cattle with higher propionate-producing rumen bacteria, such as *Succiniclasticum* and *Prevotella,* could achieve efficient feed utilization.

The rumen of the SH goat breed had a significantly higher proportion of the genus *Christensenellaceae R-7 group*, *Mogibacterium*. *Christensenellaceae R-7 group* was associated with FE, rumen digestion and absorption of nutrients, fiber digestion, and protein metabolism, and this genus produces acetic and butyric acids (Bach et al., 2019; Huang et al., 2021). Genus *Mogibacterium* was higher in the low FE group (i.e., SH). A previous study reported that *Mogibacterium* was associated with higher methane-emitting cattle (Wallace et al., 2015). Furthermore, *Mogibacterium* cannot degrade carbohydrates for energy, and it had a higher proportion in lower weight gain in steers (McLoughlin et al., 2020).

Furthermore, the rumen of the SH goats showed a numerically higher proportion than that of the family Ruminococcaceae. This family was linked to lower FE in beef cattle (Brooke et al., 2019), which supports the current findings. The function prediction of the rumen microbiome of the SH goats is enriched with hem and biotin synthesis pathways and L-arginine degradation. Hem is an important component of hemoglobin and a cofactor for many enzymes; therefore, it has an important role in oxygen transportation and various physiological processes (Yang et al., 2023). Arginine plays a key role in urea cycle regulation and protein synthesis, as ammonia is the main product of arginine degradation (Chacher et al., 2012). Biotin vitamin acts as a cofactor responsible for carbon dioxide transfer in carboxylases and was enriched in the rumen of cows with high milk yield and protein (Xue et al., 2022).

**Rumen archaea**: Methane emission from farm animals is one of the main determiners of FE. The composition of the rumen methanogens was associated with the animal breed, methane emission, and FE (Bharanidharan et al., 2018; McLoughlin et al., 2023). Archaea represent 1–2% of the microbial community and are considered the sole producer of methane in the rumen (Rabee et al., 2022; McLoughlin et al., 2023). Methane represents a 2–12% loss in FE to the host (Johnson and Johnson, 1995). PCoA analysis demonstrated (Figure 2) that the diversity of the archaeal community was influenced by the breed. The previous studies indicated that the abundance of methanogenic archaea in the rumen was associated with methane emissions and FE (Wallace et al., 2015; Zhou et al., 2010).

*Methanobrevibacter* accounted for 61.44% of the archaeal community in the SH goats, significantly more than in the ZA goats (Table 7; *p* < 0.05), which correlates with the higher predicted methane emissions in the SH goats. Meanwhile, ZA goats demonstrated a higher relative abundance of Candidatus Methanomethylophilus (64.60%) than the SH goats (18.45%). Candidatus Methanomethylophilus is an H_2_-dependent methylotrophic methanogen that derives its energy from the metabolism of methanol and methylamine (Borrel et al., 2012). This genus was associated with improved FE, higher daily gain, and lower methane emissions in different sheep breeds and Charolais steers (McLoughlin et al., 2023), which supports the current findings. Methanobrevibacter is the main methane producer in the rumen (Tapio et al., 2017). It uses hydrogen molecules, besides other substrates, such as acetate and formate, to produce methane (Jeyanathan et al., 2011). Bharanidharan et al. (2018) reported that Hanwoo steers (low efficiency) showed higher methane emissions and a higher abundance of *Methanobrevibacter* compared to Holstein steers (high efficiency), which supports our findings. The same conclusion was reported by Zhou et al. (2009) on beef steers. Previous studies reported that *Methanobrevibacter* uses acetate in methane production, leading to higher CH_4_ energy loss in low-FE animals (Nkrumah et al., 2006; Hegarty et al., 2007). Propionic acid production suppresses methane production by consuming the hydrogen from the rumen ecosystem. Furthermore, sulfur metabolism could reduce the availability of H_2_ for methane production (Zhao and Zhao, 2022). These findings explain the relationship between higher efficiency in the ZA goats and lower *Methanobrevibacter* and higher *Prevotella* as the main propionic producer in the rumen (Tapio et al., 2017; Bharanidharan et al., 2018). Thus, microbial communities in goats varied according to breed. Higher FE in the ZA goats could be attributed to higher relative abundances of lignocellulolytic bacteria that affect feed utilization positively. Moreover, a lower relative abundance of major methane-producing archaea might decline the methane energy emission (Difford et al., 2018; Gonzalez-Recio et al., 2018).

**Variance explained by markers**. FE is a quantitative trait that may be affected by a few genes with large or modest effects. Otherwise, it is affected by multiple genes with small effects (Zhang et al., 2020). In our study, we identified 26 genome windows. Each explained >0.1% of the total genetic variance explained by all windows, with a total of 1.89, 1.98, 2.39, and 1.78% of genetic variance for gross FE (G), adjusted FE (AFE), milk net energy (MNE), and FE for lactation (FEL), respectively. This suggests that the trait is likely controlled by multiple SNPs with small effects (Zhang et al., 2020). Additionally, most of the identified genes seem to have pleiotropic effects and should be considered in selective breeding programs (Sallam, 2023). Alternatively, there is a high genetic correlation between the studied traits that were undertaken to represent FE (e.g., G, AFE, MNE, and FEL). Thus, they shared common genome windows with the same positional candidate genes.

Our GWAS analysis identified multiple genomic regions that may be significantly associated with FE in Egyptian goats. The 132 megabases (Mb) on *Capra hircus* autosome (CHI) 1 was the most interesting genomic region that contributed to FE with approximately 0.26, 0.28, 0.27, and 0.3% of the total genetic variance explained for GFE, AFE, MNE, and FEL, respectively. This genomic region harbored the propionyl-CoA carboxylase subunit beta (*PCCB*) gene, which was involved in pathways related to the metabolism of water-soluble vitamins. Additionally, it is one of the two subunits of the biotin-dependent propionyl-CoA carboxylase (PCC), a mitochondrial enzyme involved in the catabolism of odd-chain fatty acids, branched-chain amino acids isoleucine, threonine, methionine, valine, and other metabolites (Wongkittichote et al., 2017).

An interesting genomic region was also located at 95 Mb on CHI11, which contributed to the genetic variance of G, AFE, MNE, and FEL with approximately 0.2, 0.16, 0.17, and 0.16%, respectively. This genomic region harbored the Golgin A1 (*GOLGA1*) gene, which is involved in vesicular trafficking at the Golgi apparatus level and endosome-to-Golgi trafficking (Wong and Munro, 2014), and the GTPase activating protein and VPS9 domains 1 (*GAPVD1*) gene, which enables GTPase activating protein binding activity and is involved in the regulation of protein transport. Additionally, the RABGAP1 (RAB GTPase activating protein 1) and DENND1A (DENN domain-containing 1A) genes are involved in pathways related to vesicle-mediated transport and trafficking regulation (Yoshimura et al., 2010; Samarelli et al., 2020). Thus, they were putative candidate genes for FE.

Two potential candidate genes were identified on CHI2 (116 Mb): the Metaxin 2 (MTX2) gene, which is involved in the transport of proteins into the mitochondrion (Del Castillo et al., 2021), and the Alkylglycerone Phosphate Synthase (AGPS) gene, which encodes a protein that catalyzes the second step of ether lipid biosynthesis (Chornyi et al., 2023). Similarly, the TMEM241 (transmembrane protein 241) was identified on CHI24 (33 Mb) and was involved in carbohydrate transport and transmembrane transport (Rodríguez et al., 2016). On CHI21 (40 Mb), the SCFD1 (Sec1 Family Domain Containing 1) gene was observed. It is involved in the regulation of protein transport (Hou et al., 2017), in addition to the Adaptor Related Protein Complex 4 Subunit Sigma 1 (AP4S1) gene, which was included in pathways related to *transporter activity* and *obsolete protein transporter activity* (Dell'Angelica et al., 1999). On CHI12 (35 Mb), the COMM Domain Containing 6 (COMMD6) gene, which is included in pathways related to protein metabolism, and TBC1D4 (TBC1 Domain Family Member 4), which is considered an insulin transport.

Similarly, the Enoyl-CoA Delta Isomerase 1 (ECI1) gene, involved in fatty acid metabolism pathways, was identified on CHI25, which spans 1 Mb. Important genes associated with fat and obesity metabolism were located on CHI18 (23 Mb), including the Iroquois Homeobox 3 (IRX3) gene, which acts as a regulator of energy metabolism and is linked to pathways involving the FTO obesity variant mechanism. Additionally, the Alpha-Ketoglutarate Dependent Dioxygenase (FTO) gene, which is known as the fat mass and obesity-associated gene, was also identified (Frayling et al., 2007).

On CHI22, which encompasses 49 Mb, the CACNA2D2 (Calcium Voltage-Gated Channel Auxiliary Subunit Alpha2delta 2) gene was found. This gene is involved in the regulation of calcium channels that control the entry of calcium ions into the cell (Bartuzi et al., 2013). Also identified on this chromosome is the TMEM115 gene, which a role in retrograde transport of proteins from the Golgi to the endoplasmic reticulum (Ong et al., 2014).

CHI8 spans 60 Mb and includes the Glucosylceramidase beta 2 (GBA2) gene, which plays a role in carbohydrate transport and metabolism (Woeste and Wachten, 2017). On CHI15, which spans 31 Mb, the STARD10 (StAR-related lipid transfer domain containing 10) gene is mapped and is predicted to be involved in lipid transport (Floris et al., 2019). The Acyl-CoA dehydrogenase family member 8 (ACAD8) gene, encoding a member of the acyl-CoA dehydrogenase family, catalyzes the dehydrogenation of acyl-CoA derivatives in fatty acids and amino acid metabolism (Zhuang et al., 2022). Additionally, the Galactosidase Beta 1 Like 3 (GLB1L3) gene, also located within this region, is predicted to be involved in carbohydrate metabolic processes (Nicoli et al., 2021).

The 36 megabases of CHI18 harbored several putative candidate genes: (1) the NRN1L (Neuritin 1-like) gene, which is involved in protein metabolism pathways (Zhang W. J. et al., 2013), and (2) the Lecithin-Cholesterol acyltransferase (LCAT) gene, which encodes an extracellular cholesterol esterifying enzyme essential for cholesterol transport (Kris-Etherton et al., 2023).

Lastly, multiple members of the SLC (solute carrier family) gene family transporters (e.g., *SLC12A4 12, SLC7A6, SLC17A9, SLCO4A1, SLC38A3,* and *SLC35G2*) were identified in most of the significant genome windows. SLC transporters participate in many important physiological functions, such as nutrient supply, and are highly expressed in different organs, including the kidney, brain, liver, gut, and heart (Zhang et al., 2019). Moreover, our gene enrichment analysis showed that the candidate genes identified in the current study were enriched in biological pathways related to the regulation of the transport process of many cellular components such as ions, lipids, organic substances, amino acids, and the general transporter activities mechanism. These findings support our hypothesis of the significant contribution of the animal genotype in FE in Egyptian goats. Thus, the findings of this study indicated that highly efficient animals have a higher representation of animal gene markers and microbial markers related to the metabolism of carbohydrates, protein, and lipids. Therefore, this information could be a useful aid in selective breeding programs that aim at improving FE in goats.

## Conclusion

The ZA goats showed higher FE compared to the SH goats. These differences in FE may result from variations in rumen microbiota, with ZA goats demonstrating enhanced fiber utilization and reduced methane emissions. Furthermore, association analysis revealed 26 genome windows containing several putative candidate genes that significantly contribute to FE traits in Egyptian goats. These genes are involved in pathways related to cellular component metabolism and transport, making them promising candidates for enhancing FE in Egyptian goats. In addition, variations were observed between the goat breeds in terms of milk protein content and certain milk fatty acids.

Future studies should leverage genetic markers and microbial insights to improve goat breeding, enhance FE, and optimize milk quality in arid regions while also aiming to also reduce methane emissions.

## Data Availability

The original contributions presented in the study are publicly available. This data can be found at: https://www.ncbi.nlm.nih.gov/sra/PRJNA1047779. The host genome datasets generated and/or analyzed during the current study are available for the corresponding breeds in the OSF repository using the following link: https://osf.io/7q6j8/ and can be downloaded upon request from the corresponding author.

## Footnotes

^1^ http://www.amdis.net/

^2^ https://www.ensembl.org/biomart/martview
